# Changes of Body Mass Index in Relation to Mortality: Results of a Cohort of 42,099 Adults

**DOI:** 10.1371/journal.pone.0084817

**Published:** 2014-01-08

**Authors:** Jochen Klenk, Kilian Rapp, Hanno Ulmer, Hans Concin, Gabriele Nagel

**Affiliations:** 1 Institute of Epidemiology and Medical Biometry, Ulm University, Ulm, Germany; 2 Clinic for Geriatric Rehabilitation, Robert-Bosch Hospital, Stuttgart, Germany; 3 Department of Medical Statistics, Informatics and Health Economics, Medical University, Innsbruck, Austria; 4 Agency for Preventive and Social Medicine, Bregenz, Austria; Peking Union Medical College, China

## Abstract

**Background:**

High Body-Mass-Index (BMI) is associated with increased all-cause mortality, but little is known about the effect of short- and long-term BMI change on mortality. The aim of the study was to determine how long-term weight change affects mortality.

**Methods and findings:**

Within a population-based prospective cohort of 42,099 Austrian men and women (mean age 43 years) with at least three BMI measurements we investigated the relationship of BMI at baseline and two subsequent BMI change intervals of five years each with all-cause mortality using Cox proportional Hazard models. During median follow-up of 12 years 4,119 deaths were identified. The lowest mortalities were found in persons with normal weight or overweight at baseline and stable BMI over 10 years. Weight gain (≥0.10 kg/m^2^/year) during the first five years was associated with increased mortality in overweight and obese people. For weight gain during both time intervals mortality risk remained significantly increased only in overweight (Hazard Ratio (HR): 1.39 (95% confidence interval: 1.01; 1.92)) and obese women (1.85 (95% confidence interval: 1.18; 2.89)). Weight loss (< −0.10 kg/m^2^/year) increased all-cause mortality in men and women consistently. BMI change over time assessed using accepted World Health Organisation BMI categories showed no increased mortality risk for people who remained in the normal or overweight category for all three measurements. In contrast, HRs for stable obese men and women were 1.57 (95% CI: 1.31; 1.87) and 1.46 (95% CI: 1.25; 1.71) respectively.

**Conclusion:**

Our findings highlight the importance of weight stability and obesity avoidance in prevention strategy.

## Introduction

Over the last decades, the prevalence of overweight and obesity has increased in most industrialized countries and reached alarming dimensions [1]. Many diseases are linked to excess body-weight such as type II diabetes, coronary heart disease, stroke or various types of cancer [2]–[5]. Several studies have shown a relationship between obesity and increased all-cause mortality [6]–[11]. For overweight, little to no increased mortality risk has been reported [6]–[11].

BMI is a common clinical measure for overweight and obesity. Although many previously published studies have been limited to a single BMI measurement, some studies suggest that changes in BMI over time may be of greater significance to public health. However, the impact of BMI change on all-cause mortality remains controversial.

Several studies found a relationship between BMI loss and elevated all-cause mortality [12]–[18]. The observed associations between BMI gain and all-cause mortality are inconsistent. Some authors reported positive associations [12], [13], [15], [19]–[21], while others did not find any relationship [14], [16], [17]. Data on the effects of long- term weight changes in population-based cohorts are sparse.

Methodological problems like reverse causation or lack of knowledge about the intention of weight loss make causality difficult to interpret [22], [23]. In addition, weight change may be associated with pre-existing disease or subclinical conditions.

In order to clarify the effect of long-term BMI change patterns, we conducted a prospective study to analyse the effect of BMI at baseline and two subsequent BMI change intervals of five years each on all-cause mortality in a cohort of 42,099 Austrian men and women.

## Methods

### Study Population

The Vorarlberg Health Monitoring & Prevention Program (VHM&PP) is a population-based risk factor surveillance program in Vorarlberg, the westernmost province of Austria. The program is administrated by the Agency of Social and Preventive Medicine (aks). All adults (aged ≥19 years) within the province were invited to participate. Enrolment is voluntary and costs for one examination per year are covered by the participant’s compulsory health insurance. The screening examinations take place in the practices of local physicians according to a standard protocol. Anthropometric measures were carried out with participants wearing light indoor clothes and no shoes. Most of the participants have two or more registered visits with varying time interval between them. Details of the programme and characteristics of the study population have been previously described [4], [9], [24]. Between January 1985 and June 2005, 185,316 adult Vorarlberg residents were enrolled in the VHM&PP Study Cohort after signing informed consent. This corresponds to more than 60% of the general population in the eligible age range.

**Figure 1:**
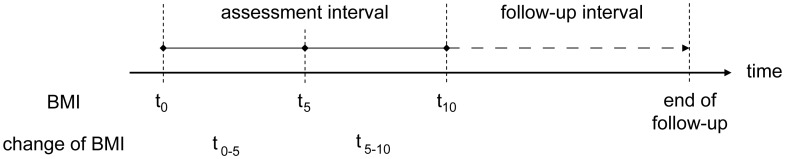
Time line of BMI assessments, BMI change intervals and subsequent follow up.

In order to assess two subsequent BMI change intervals, three measurement time points (t) were defined: baseline (t_0_), year 5 (t_5_), and year 10 (t_10_) (**Figure 1**). For t_5_ or t_10,_ data within the year after or the year before wereaccepted. Change of BMI was calculated between t_0_ and t_5_ (t_0–5_) and the subsequent time interval t_5_ and t_10_ (t_5–10_). In total, 43,233 men and women with complete data on height, weight and smoking status were available. No information was available regarding participant morbidity status at baseline. Therefore, due to increased probability of pre-existing disease, participants who were underweight (BMI<18.5 kg/m^2^) at baseline were excluded (n = 1,134).

### Classification of BMI at Baseline and Changes Over Time

Height and weight were measured by medical staff during the VHM&PP physical examinations. BMI at t_0_, t_5_, and t_10_ was classified according to World Health Organisation (WHO) guidelines normal (18.5–24.9 kg/m^2^), overweight (25.0–29.9 kg/m^2^), and obese (≥30.0 kg/m^2^). Normal BMI was defined as reference category for statistical analyses. BMI change per year was calculated for each of the considered time intervals. Three categories were defined: weight loss (<−0.10 kg/m^2^/year), stable weight (−0.10 to 0.09 kg/m^2^/year), and weight gain (≥0.10 kg/m^2^/year). To analyse the patterns of two subsequent BMI change intervals, 27 different combinations of baseline BMI and BMI change were considered. People with normal weight and no weight change during both intervals was used as reference category.

### Endpoint

All-cause mortality served as endpoint for all analyses. Follow-up started after the last BMI measurement at t_10_. Death information was provided by the Vorarlberg Death Registry and was linked to the cohort data. All deaths were identified from death certificates and were confirmed by trained physicians only. In case of unclear causes of death, autopsies were performed (23.3% of all deaths in Austria in 2002) [25]. Observation time started 01.01.1985 and participants were censored by December 31, 2009.

### Covariates

All analyses were adjusted for smoking by including information on current smoking and former smoking in the model. Persons with missing smoking data were classified as never smokers because the baseline questionnaires did not differentiate between never-smokers and participants with missing values [9]. Smoking information from follow-up visits was available for most of the participants to complement the baseline smoking status. The category of never-smokers contains therefore only a low percentage of misclassified smokers and ex-smokers. Due to the potential for over-adjustment, we did not adjust for assumed intermediate steps in the causal pathway [11].

### Statistical Analysis

Hazard rate ratios (HR) and 95% confidence intervals (95% CI) were used to estimate the effect of baseline BMI, combinations of baseline BMI and the first BMI change interval (t_0–5_), as well as, combinations of baseline BMI and both subsequent BMI change intervals (t_0–5_+ t_5–10_) on all-cause mortality. Stratification according to baseline BMI was performed in order to explore possible effect modification.

Furthermore, the pattern of BMI over time was assessed for each participant by comparing changes in BMI category (as defined by the WHO) from t_0_ to t_5_ to t_10_. (e.g. normal weight – overweight – normal weight). For t_5_ and t_10_ the category underweight was included. The relationship of these patterns with all-cause mortality was estimated by Cox proportional hazards models and was displayed as Hazard ratios (HR).

All analyses were performed separately for men and women. Models included age (in single years) in the strata statement and adjustment for smoking status. The calculations were performed using SAS 9.2.

## Results

The final dataset included 17,772 men and 24,327 women with a mean age at baseline of 42.9 (SD = 13.1) and 43.0 (SD = 13.5) years, respectively (**Table 1**). Ever smoking was more prevalent in men (44.1%) than in women (26.7%). During a median follow-up of 11.8 years 2,053 deaths were registered in males and 2,066 in females. At baseline prevalence rates of overweight and obesity were 42.2% and 7.8% in men and 26.2% and 9.5% in women, respectively.

**Table 1:** Characteristics of study population: VHM&PP Study Cohort 1985–2009.

	Men		Women	
n	17,772		24,327	
age [years] at t_0_, mean (sd)	42.9 (13.1)		43.0 (13.5)	
BMI [kg/m^2^] at t_0_, mean (sd)	25.3 (3.17)		24.4 (4.05)	
18.5–24.9 kg/m^2^, n (%)	8,895 (50.1%)		15,659 (64.4%)	
25.0–29.9 kg/m^2^, n (%)	7,496 (42.2%)		6,365 (26.2%)	
30.0–35.0 kg/m^2^, n (%)	1,381 (7.8%)		2,303 (9.5%)	
	**t_0–5_**	**t_5–10_**	**t_0–5_**	**t_5–10_**
assessment interval [years], mean (sd)	5.2 (0.79)	4.9 (1.03)	5.2 (0.75)	4.9 (1.00)
Change of BMI [kg/m^2^/year], mean (sd)	0.10 (0.31)	0.09 (0.34)	0.13 (0.39)	0.12 (0.41)
<−0.10 kg/m^2^/year, n (%)	3,760 (21.2%)	4,025 (22.7%)	5,352 (22.0%)	5,621 (23.1%)
−0.10–0.09 kg/m^2^/year, n (%)	5,527 (31.1%)	5,372 (30.2%)	6,635 (27.3%)	6,275 (25.8%)
≥0.10 kg/m^2^/year, n (%)	8,485 (47.7%)	8,375 (47.1%)	12,340 (50.7%)	12,431 (51.1%)
Ever smoker, n (%)	7,835 (44.1%)		6,487 (26.7%)	
years of follow-up, median (Q1–Q3)	11.3 (8.1–13.4)		12.1 (9.1–13.7)	
number of deaths, n (%)	2,053 (11.6%)		2,066 (8.5%)	

In men (**Table 2**) and women (**Table 3**) a dose-response relationship was seen with increasing baseline BMI for all-cause mortality. Compared to normal weight, statistically significant HRs for overweight and obesity were 1.13 (95% CI: 1.03; 1.24) and 1.50 (95% CI: 1.30; 1.73) in men and 1.17 (95% CI: 1.06; 1.29) and 1.52 (95% CI: 1.34; 1.72) in women, respectively.

**Table 2:** Effect of change of BMI between t_0–5_ and t_0–5_+ t_5–10_ on all cause-mortality in men stratified for baseline BMI: VHM&PP Study Cohort 1985–2009.

Baseline				t_0–5_ *				t_5–10_ *			
Baseline BMI	N (%)	Fatal events	HR (95% CI)§	Pattern	N (%)	Fatal events	HR (95% CI) §	Pattern	N (%)	Fatal events	HR (95% CI) §
								/	160 (0.9)	38	1.56 (1.05; 2.32)
				/	1,329 (7.5)	170	1.14 (0.93; 1.39)	–	406 (2.3)	57	1.23 (0.87; 1.73)
								/	763 (4.3)	75	1.27 (0.93; 1.73)
								/	448 (2.5)	79	1.84 (1.35; 2.51)
18.5–24.9 kg/m^2^	8,895 (50.1)	757	1.00 (ref)#	–	2,894 (16.3)	250	1.00 (ref)#	–	1,008 (5.7)	85	1.00 (ref)#
								/	1,438 (8.1)	86	0.95 (0.70; 1.28)
								/	1,159 (6.5)	139	1.58 (1.20; 2.08)
				/	4,672 (26.3)	337	1.12 (0.95; 1.32)	–	1,534 (8.6)	100	1.11 (0.83; 1.48)
								/	1,979 (11.1)	98	1.14 (0.85; 1.53)
								/	320 (1.8)	83	1.86 (1.37; 2.53)
				/	1,918 (10.8)	331	1.31 (1.11; 1.55)	–	483 (2.7)	83	1.32 (0.98; 1.79)
								/	1,115 (6.3)	165	1.47 (1.13; 1.92)
								/	521 (2.9)	103	1.75 (1.31; 2.35)
25.0–29.9 kg/m^2^	7,496 (42.2)	1,039	1.13 (1.03; 1.24)	–	2,335 (13.1)	340	1.17 (0.996; 1.39)	–	687 (3.9)	93	1.15 (0.86; 1.55)
								/	1,127 (6.3)	144	1.26 (0.96; 1.65)
								/	984 (5.5)	156	1.66 (1.27; 2.17)
				/	3,243 (18.3)	368	1.18 (1.002; 1.39)	–	946 (5.3)	93	1.09 (0.81; 1.46)
								/	1,313 (7.4)	119	1.27 (0.96; 1.68)
								/	129 (0.7)	34	1.96 (1.31; 2.96)
				/	513 (2.9)	111	1.67 (1.33; 2.09)	–	104 (0.6)	28	2.08 (1.35; 3.19)
								/	280 (1.6)	49	1.80 (1.26; 2.57)
								/	93 (0.5)	26	2.12 (1.36; 3.30)
≥30.0 kg/m^2^	1,381 (7.8)	257	1.50 (1.30; 1.73)	–	298 (1.7)	59	1.50 (1.13; 2.00)	–	74 (0.4)	11	1.32 (0.70; 2.48)
								/	131 (0.7)	22	1.60 (0.995; 2.56)
								/	211 (1.2)	41	2.34 (1.61; 3.40)
				/	570 (3.2)	87	1.63 (1.28; 2.09)	–	130 (0.7)	23	1.64 (1.03; 2.61)
								/	229 (1.3)	23	1.51 (0.95; 2.40)

change of BMI (<−0.10 kg/m^2^/year, −0.10–0.09 kg/m^2^/year, ≥0.10 kg/m^2^/year) between baseline and year 5 (t_0–5_) and between year 5 and 10 (t_5–10_).

Hazard rate ratio (HR) and 95% confidence interval (95% CI) adjusted for smoking status and stratified for age.

^#^ reference category.

**Table 3:** Effect of change of BMI between t_0–5_ and t_0–5_+ t_5–10_ on all cause-mortality in women stratified for baseline BMI: VHM&PP Study Cohort 1985–2009.

Baseline				t_0–5_ *				t_5–10_ *			
Baseline BMI	N (%)	Fatal events	HR (95% CI) §	Pattern	N (%)	Fatal events	HR (95% CI) §	Pattern	N (%)	Fatal events	HR (95% CI) §
								/	331 (1.4)	65	2.54 (1.80; 3.59)
				/	2,811 (11.6)	252	1.25 (1.05; 1.48)	–	725 (3.0)	67	1.60 (1.14; 2.25)
								/	1,755 (7.2)	120	1.34 (0.99; 1.80)
								/	793 (3.3)	88	1.78 (1.30; 2.45)
18.5–24.9 kg/m^2^	15,659 (64.4)	913	1.00 (ref)#	–	4,614 (19.0)	284	1.00 (ref)#	–	1,436 (5.9)	68	1.00 (ref)#
								/	2,385 (9.8)	128	1.22 (0.90; 1.63)
								/	2,049 (8.4)	134	1.47 (1.09; 1.97)
				/	8,234 (33.9)	377	0.96 (0.83; 1.13)	–	2,304 (9.5)	113	1.21 (0.89; 1.63)
								/	3,881 (16.0)	130	1.06 (0.79; 1.42)
								/	343 (1.4)	87	2.14 (1.55; 2.95)
				/	1,699 (7.0)	282	1.30 (1.10; 1.54)	–	335 (1.4)	54	1.42 (0.99; 2.04)
								/	1,021 (4.2)	141	1.58 (1.18; 2.12)
								/	393 (1.6)	70	1.56 (1.12; 2.19)
25.0–29.9 kg/m^2^	6,365 (26.2)	806	1.17 (1.06; 1.29)	–	1,586 (6.5)	209	1.11 (0.92; 1.33)	–	385 (1.6)	53	1.31 (0.91; 1.88)
								/	808 (3.3)	86	1.39 (1.01; 1.92)
								/	954 (3.9)	137	1.88 (1.40; 2.52)
				/	3,080 (12.7)	315	1.24 (1.05; 1.45)	–	675 (2.8)	59	1.25 (0.88; 1.78)
								/	1,451 (6.0)	119	1.49 (1.10; 2.01)
								/	247 (1.0)	74	3.13 (2.24; 4.37)
				/	842 (3.5)	168	1.85 (1.52; 2.24)	–	137 (0.6)	32	2.37 (1.55; 3.62)
								/	458 (1.9)	62	1.85 (1.31; 2.61)
								/	139 (0.6)	31	2.24 (1.46; 3.44)
≥30.0 kg/m^2^	2,303 (9.5)	347	1.52 (1.34; 1.72)	–	435 (1.8)	70	1.38 (1.06; 1.80)	–	92 (0.4)	12	1.10 (0.59; 2.03)
								/	204 (0.8)	27	1.85 (1.18; 2.89)
								/	372 (1.5)	49	1.94 (1.34; 2.81)
				/	1,026 (4.2)	109	1.40 (1.12; 1.75)	–	186 (0.8)	20	1.71 (1.03; 2.83)
								/	468 (1.9)	40	1.66 (1.12; 2.46)

change of BMI (<−0.10 kg/m^2^/year, −0.10–0.09 kg/m^2^/year, ≥0.10 kg/m^2^/year) between baseline and year 5 (t_0–5_) and between year 5 and 10 (t_5–10_).

Hazard rate ratio (HR) and 95% confidence interval (95% CI) adjusted for smoking status and stratified for age.

^#^ reference category.

Considering the combination of baseline BMI and the first BMI change interval (t_0–5_) no statistically significant increased mortality was seen in stable overweight persons but in stable obese men (**Table 2** **)** and women (**Table 3**). Gaining BMI was associated with significant higher mortality in overweight, as well as, obese men and women. Losing weight increased HRs significantly in all BMI categories except for normal weight men.

Including the subsequent BMI interval (t_5–10_), a more detailed picture was seen. BMI stability in overweight and obese persons during t_0–5_ and t_5–10_ did not significantly increase mortality compared to persons maintaining normal weight. Gaining BMI after weight stability in the first interval increased mortality only in women who were overweight (1.39 (95% CI: 1.01; 1.92)) or obese (1.85 (95% CI: 1.18; 2.89)) at baseline.

A continuous increase of BMI in both time intervals between t_0_ and t_10_ showed a dose-response relationship with mortality for increasing baseline BMI in men but estimates were not statistically significant. In women continuous weight gain was not associated with death for normal weight at baseline, whereas, HRs for overweight and obesity were 1.49 (95% CI: 1.10; 2.01) and 1.66 (95% CI 1.12; 2.46), respectively. Gaining BMI after a weight loss period showed a significant elevated mortality in overweight and obese but not in normal weight persons. Estimates for men and women were 1.47 (95% CI: 1.13; 1.92) and 1.80 (95% CI: 1.26; 2.57) as well as 1.58 (95% CI: 1.18; 2.12) and 1.85 (95% CI: 1.31; 2.61), respectively. Losing weight between t_5_ and t_10_ showed the largest HRs in all categories independent of the previous pattern (t_0–5_). A decrease of BMI between t_0_ and t_5_ with a subsequent period of BMI stability increased estimates in both genders but were only statistically significant in obese men and women who were normal weight or obese.

For men and women increase of BMI was the most common pattern in both assessment intervals (about 50%) while BMI stability (about 30%) and loss of BMI (about 20%) was less prevalent (**Table 2** **and** **3**). The proportion of participants with stable BMI over both observation intervals (t_0–5_ and t_5–10_) by BMI category (normal, overweight, obese) was 11.3%, 9.2%, and 5.4% for men and 9.2%, 6.0%, and 4.0% for women related to the number of persons within each BMI category.

**Figure 2:**
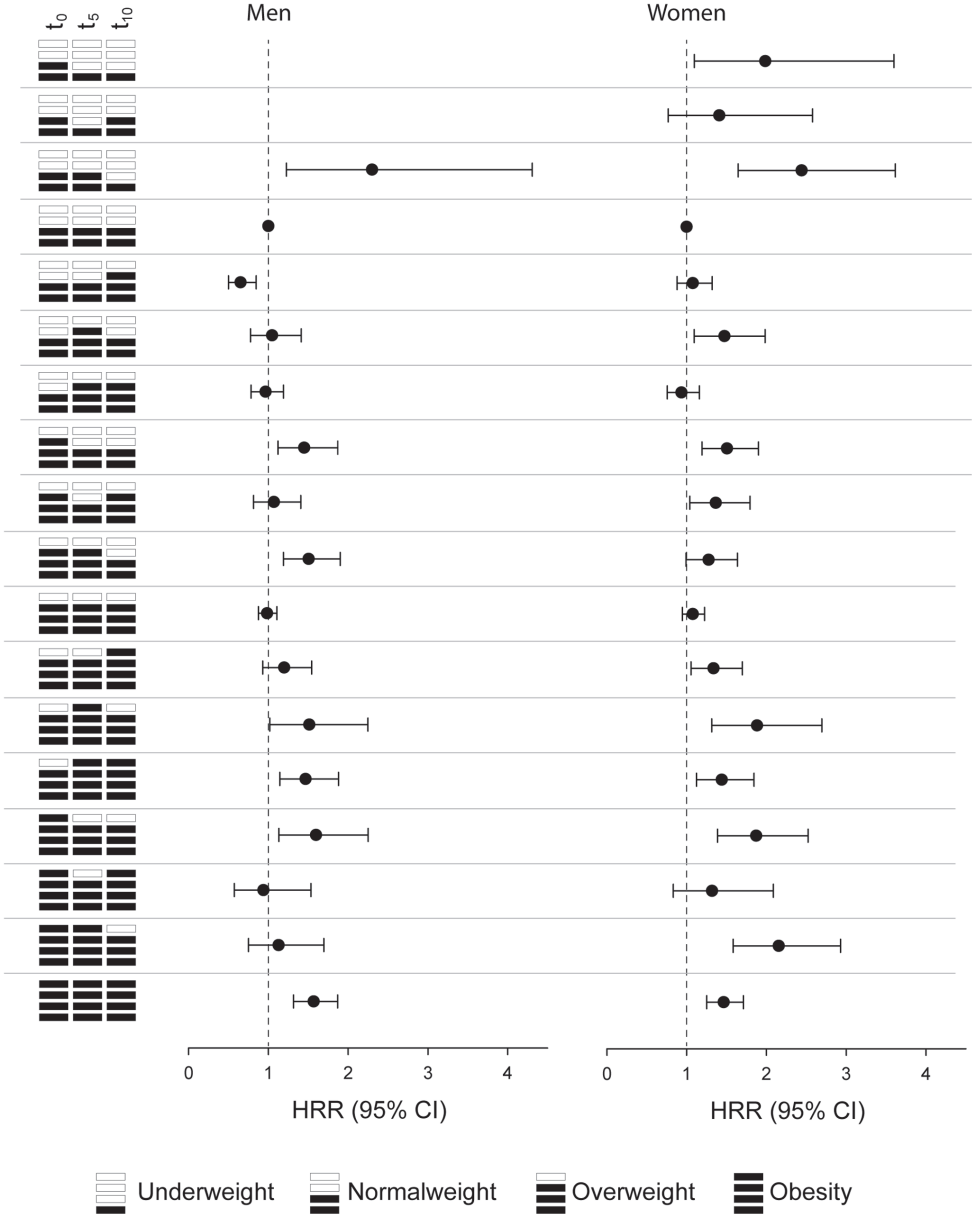
Effect of different patterns of BMI at t_0_, t_5_, and t_10_ on all cause-mortality among men and women in the VHM&PP Study Cohort 1985–2009.

**Figure 2** shows the effects of different patterns of WHO BMI categories at t_0_, t_5_, and t_10_ on all-cause mortality. In people maintaining normal weight and overweight between t_0_ and t_10_ no increased mortality risk was found. In contrast HRs for obese men and women were 1.57 (95% CI: 1.31; 1.87) and 1.46 (95% CI: 1.25; 1.71), respectively. Changing from normal weight to overweight did not increase mortality for either gender. For men, the HRs decreased even to 0.65 (95% CI: 0.50; 0.85), when the BMI category increased during the last interval. Change from overweight to obesity in the first interval increased mortality risk significantly in both genders. Change from overweight to obesity in the second interval increased mortality risk in women only. Decreases in BMI category or BMI cycling showed either no effect or elevated HRs.

## Discussion

### Principal Findings

With the large number of participants with repeated anthropometric measurements and the reliable link to the local death registry we were able to study the association between BMI change during two subsequent time intervals of five years and all-cause mortality. Overall, the lowest risks were seen in normal weight and overweight subjects with stable BMI over time. Weight gain was associated with mortality in overweight and obese persons, although not statistically significant in all cases. Weight loss increased HRs in men and women consistently.

Our findings concerning the association between BMI at baseline and all-cause mortality are consistent with a previous analysis of a larger sample from the VHM&PP cohort [9] and with those from other large cohorts [6]–[8], [10], [11] This indicates that selection bias in the present subsample is unlikely and that the baseline mortality risk of BMI is comparable with other populations.

Overall, long-term weight stability was prevalent in 30% of our cohort. Our finding that stable BMI in normal weight and overweight participants showed the lowest risk for all-cause mortality is consistent with results of a recent review [26]. A stable BMI may reflect a more balanced lifestyle with less exposure to unhealthy behaviors such as smoking or physical inactivity [27]. However, weight gain was the most prevalent pattern in our cohort. A stable BMI during both time intervals was seen only in about 10% of all participants with normal weight at baseline. The proportion became considerable smaller by increasing BMI category. Similar findings were seen in the literature [26].

The observed positive relationships between weight loss and increased mortality in our study is consistent with several previous studies [12]–[18] However, the interpretation is not straightforward. Most studies accounted for health status, but information may have been incomplete or underlying diseases might not have been detected [18]. Furthermore, the majority of investigations did not differentiate between intentional and unintentional weight loss [18]. In our cohort, there was also no information about morbidity or intention of weight loss. However, in order to investigate the potential for unintended weight loss due to pre-existing diseases, we conducted sensitivity analyses excluding death three years after the last BMI measurement. The patterns did not change substantially (data not shown). In addition, we analysed weight loss with more detail than previous studies by investigating two consecutive time intervals of 5 years. Consequently, we observed that weight loss was associated with higher mortality regardless of weight change in the other time period. Nevertheless, our findings related to weight reductions and all-cause mortality should be interpreted with caution. Overall, long-term unintentional weight loss appears as an important health indicator, which needs to be further explored in age and disease specific context.

We found that continuous weight gain over both time intervals (t_0–5_+ t_5–10_) was associated with increased all-cause mortality in overweight and obese persons, although not statistically significant in men. The majority of previous studies also reported a positive relationship between increase in body weight and mortality [12], [13], [15], [19]–[21], however, several studies did not find any relation [14], [16], [17] The varying results may be due to different approaches for assessing weight change. Weight and height were either recalled from the past, self-reported or measured. Our study used only valid height and weight measurements assessed by trained medical staff. Furthermore, the number of measurements and the length of follow-up varied considerably between studies. Data from the Framingham heart study showed that the length of follow-up influenced the association of BMI and mortality [28].

Another issue concerning the literature on weight change and mortality is the lack of accepted cut-points to define categories of weight change. Consequently, weight change categories differed between studies. In some studies, the highest category of weight gain included participants with only moderate weight gain (≥5 kg within 10 years) [12], [14] Conversely, in one North-American investigation, the highest category of weight gain comprised women gaining more than 40 kg within a varying assessment period of 12–37 years [15].

In contrast to many other studies that define absolute weight gain as the primary outcome, our study benefits from the use of relative measures (change in kg/m^2^/year). Weight stability in our categorization refers to ±2.6 kg in a person of 1.60 meters of height and ±3.4 kg in a person of 1.85 meters over a period of 10 years and for weight gain to ≥12.8 kg and ≥17.1 kg, respectively.

### Strengths and Weaknesses of the Study

A major strength of our study is that we could analyze long-term BMI change patterns of two subsequent 5-year-intervals on an individual level in a very large study population with more than 10 years of follow-up. Additionally, we used widely accepted BMI categories specified by the WHO whereby results can be used for public health recommendations. Finally, as the average age within our study population was 43 years, our cohort should have consisted of few chronically ill individuals. Therefore, we believe that unintentional weight loss as result of chronic illness was minimal.

Using BMI as a measure of overweight and obesity may be considered as a limitation. BMI reflects different body compartments and is not informative concerning the fat and lean body mass distribution [29]. Smoking affects the BMI and the body fat distribution [30], which may have confounded the relationship between BMI change and over-all mortality. However, exclusion of ever smokers revealed similar patterns, but increased estimates in obese never-smoking men (Table S1 and S2 in File S1). The ratio between fat and lean body mass is a result of physiology, aging, energy intake, and physical activity [31], [32] Aging is characterized by loss of subcutaneous fat and ectopic fat deposition [31]. However, due to the high correlation of BMI with body fat, BMI is generally considered a reasonable measure of obesity in epidemiological studies. We used BMI between 18.5 and 24.9 kg/m^2^ as reference category. A recent meta-analysis including 2.88 million individuals showed no mortality increase for BMI values less than 35 kg/m^2^ [11]. This is consistent with our finding that weight stability in overweight persons did not increase mortality risk. There could be an age-dependent range of BMI levels associated with equivalent risk of death [33]. Thus, more refined measurements of the fat-lean body mass ratio may be necessary to further clarify the association between BMI and mortality in relation to age [7]. Finally, despite the large sample size results did not reach statistical significance in some cases. However, dose-response relationships were seen. Unfortunately we do not have information on further potential risk factors such as comorbidities. Concerning generalizability it has to be noted that participants voluntary participated in the study and had to survive at least 10 years due to our study design. Therefore, mortality of this cohort might be lower compared to the general population.

### Conclusions

In summary, our data indicate that obesity should be avoided and that weight gain in overweight and obese persons is a risk factor for all-cause mortality. The results regarding weight loss and mortality, though consistent in all time intervals, should be interpreted with caution. The lowest mortality risks were seen with normal weight and overweight people maintaining BMI over 10 years. Therefore, our findings highlight weight stability within these BMI ranges as an important public health prevention strategy.

### Ethical Approval

Ethical approval the Vorarlberg ethic commission EK-Nr. 2006-6/2.

## Supporting Information

File S1 Supporting tables. Table S1, Effect of change of BMI between t0–5 and t0–5+ t5–10 on all cause-mortality in never-smoking men stratified for baseline BMI: VHM&PP Study Cohort 1985–2009. Table S2, Effect of change of BMI between t_0–5_ and t_0–5_+t_5–10_ on all cause-mortality in never-smoking women stratified for baseline BMI: VHM&PP Study Cohort 1985–2009.
(DOC)
