# Supplementary material for: Changes of Body Mass Index in Relation to Mortality: Results of a Cohort of 42,099 Adults
Source: PLoS One. 2014 Jan 8;9(1):e84817. doi: 10.1371/journal.pone.0084817 (PMC3885599; doi:10.1371/journal.pone.0084817)
Supplement: File S1 — Supporting tables. Table S1, Effect of change of BMI between t0–5 and t0–5+ t5–10 on all cause-mortality in never-smoking men stratified for baseline BMI: VHM&PP Study Cohort 1985–2009. Table S2, Effect of change of BMI between t0–5 and t0–5+t5–10 on all cause-mortality in never-smoking women stratified for baseline BMI: VHM&PP Study Cohort 1985–2009. (DOC) [file pone.0084817.s001.doc]

**Table S1**: Effect of change of BMI between t_0-5_ and t_0-5_ + t_5-10_ on all cause-mortality **in never-smoking men** stratified for baseline BMI: VHM&PP Study Cohort 1985-2009

| Baseline |  |  |  |  | t_0-5_* |  |  |  |  | t_5-10_* |  |  |  |
| --- | --- | --- | --- | --- | --- | --- | --- | --- | --- | --- | --- | --- | --- |
| Baseline BMI | N (%) | Fatal events | HRR (95% CI)^§^ |  | Pattern | N (%) | Fatal events | HRR (95% CI) ^§^ |  | Pattern | N (%) | Fatal events | HRR (95% CI) ^§^ |
|  |  |  |  |  |  |  |  |  |  | ̷⁄ | 86 (0.9) | 16 | 1.35 (0.75; 2.43) |
|  |  |  |  |  | ⁄ | 748 (7.5) | 79 | 1.06 (0.80; 1.41) |  | ─ | 248 (2.5) | 29 | 1.24 (0.77; 2.01) |
|  |  |  |  |  |  |  |  |  |  | ⁄ | 414 (4.2) | 34 | 1.12 (0.72; 1.76) |
|  |  |  |  |  |  |  |  |  |  | ̷⁄ | 253 (2.6) | 40 | 1.75 (1.13; 2.70) |
| 18.5 – 24.9 kg/m^2^ | 5,170 (52.0) | 362 | 1.00^#^ |  | ─ | 1,755 (17.7) | 132 | 1.00^#^ |  | ─ | 623 (6.3) | 46 | 1.00^#^ |
|  |  |  |  |  |  |  |  |  |  | ⁄ | 879 (8.9) | 46 | 0.97 (0.64; 1.46) |
|  |  |  |  |  |  |  |  |  |  | ̷⁄ | 645 (6.5) | 65 | 1.50 (1.02; 2.21) |
|  |  |  |  |  | ⁄ | 2,667 (26.8) | 151 | 1.05 (0.83; 1.33) |  | ─ | 912 (9.2) | 46 | 0.98 (0.65; 1.48) |
|  |  |  |  |  |  |  |  |  |  | ⁄ | 1,110 (11.2) | 40 | 1.07 (0.69; 1.64) |
|  |  |  |  |  |  |  |  |  |  | ̷⁄ | 174 (1.8) | 40 | 1.74 (1.12; 2.69) |
|  |  |  |  |  | ⁄ | 1,006 (10.1) | 158 | 1.42 (1.12; 1.80) |  | ─ | 270 (2.7) | 49 | 1.52 (1.01; 2.28) |
|  |  |  |  |  |  |  |  |  |  | ⁄ | 562 (5.7) | 69 | 1.61 (1.11; 2.35) |
|  |  |  |  |  |  |  |  |  |  | ̷⁄ | 307 (3.1) | 61 | 1.94 (1.32; 2.87) |
| 25.0 – 29.9 kg/m^2^ | 4,090 (41.2) | 527 | 1.29 (1.12; 1.48) |  | ─ | 1,346 (13.6) | 184 | 1.24 (0.99; 1.56) |  | ─ | 400 (4.0) | 47 | 1.14 (0.75; 1.71) |
|  |  |  |  |  |  |  |  |  |  | ⁄ | 639 (6.4) | 76 | 1.29 (0.89; 1.87) |
|  |  |  |  |  |  |  |  |  |  | ̷⁄ | 524 (5.3) | 78 | 1.92 (1.33; 2.78) |
|  |  |  |  |  | ⁄ | 1,738 (17.5) | 185 | 1.35 (1.07; 1.69) |  | ─ | 523 (5.3) | 48 | 1.19 (0.79; 1.79) |
|  |  |  |  |  |  |  |  |  |  | ⁄ | 691 (7.0) | 59 | 1.45 (0.98; 2.15) |
|  |  |  |  |  |  |  |  |  |  | ̷⁄ | 63 (0.6) | 20 | 2.97 (1.74; 5.07) |
|  |  |  |  |  | ⁄ | 243 (2.5) | 53 | 1.95 (1.40; 2.72) |  | ─ | 50 (0.5) | 12 | 1.84 (0.97; 3.51) |
|  |  |  |  |  |  |  |  |  |  | ⁄ | 130 (1.3) | 21 | 1.95 (1.14; 3.34) |
|  |  |  |  |  |  |  |  |  |  | ̷⁄ | 44 (0.4) | 10 | 2.07 (1.03; 4.16) |
| ≥30.0 kg/m^2^ | 677 (6.8) | 128 | 1.92 (1.55; 2.36) |  | ─ | 143 (1.4) | 30 | 1.98 (1.32; 2.96) |  | ─ | 30 (0.3) | 4 | 1.57 (0.56; 4.40) |
|  |  |  |  |  |  |  |  |  |  | ⁄ | 69 (0.7) | 16 | 2.64 (1.48; 4.71) |
|  |  |  |  |  |  |  |  |  |  | ̷⁄ | 115 (1.2) | 23 | 2.89 (1.74; 4.79) |
|  |  |  |  |  | ⁄ | 291 (2.9) | 45 | 2.01 (1.42; 2.83) |  | ─ | 71 (0.7) | 10 | 1.62 (0.80; 3.28) |
|  |  |  |  |  |  |  |  |  |  | ⁄ | 105 (1.1) | 12 | 2.10 (1.10; 3.98) |

* change of BMI (<-0.10 kg/m²/year, -0.10 – 0.09 kg/m²/year, ≥0.10 kg/m²/year) between baseline and year 5 (t_0-5_) and between year 5 and 10 (t_5-10_)

^§^ Hazard rate ratio (HRR) and 95% confidence interval (95% CI) adjusted for smoking status and stratified for age

^#^ reference category

**Table S 2**: Effect of change of BMI between t_0-5_ and t_0-5_ + t_5-10_ on all cause-mortality **in never-smoking women** stratified for baseline BMI: VHM&PP Study Cohort 1985-2009

| Baseline | | | |  | t_0-5_* | | | |  | t_5-10_* | | | |
| --- | --- | --- | --- | --- | --- | --- | --- | --- | --- | --- | --- | --- | --- |
| Baseline BMI | N (%) | Fatal events | HRR (95% CI) ^§^ |  | Pattern | N (%) | Fatal events | HRR (95% CI) ^§^ |  | Pattern | N (%) | Fatal events | HRR (95% CI) ^§^ |
|  |  |  |  |  |  |  |  |  |  | ̷⁄ | 225 (1.3) | 57 | 2.67 (1.83; 3.89) |
|  |  |  |  |  | ⁄ | 1,877 (10.5) | 206 | 1.27 (1.05; 1.54) |  | ─ | 501 (2.8) | 55 | 1.54 (1.05; 2.24) |
|  |  |  |  |  |  |  |  |  |  | ⁄ | 1,151 (6.5) | 94 | 1.32 (0.95; 1.86) |
|  |  |  |  |  |  |  |  |  |  | ̷⁄ | 563 (3.2) | 72 | 1.74 (1.22; 2.48) |
| 18.5 – 24.9 kg/m^2^ | 11,070 ( 62.1) | 721 | 1.00^#^ |  | ─ | 3,351 (18.8) | 222 | 1.00^#^ |  | ─ | 1,049 (5.9) | 55 | 1.00^#^ |
|  |  |  |  |  |  |  |  |  |  | ⁄ | 1,739 (9.8) | 95 | 1.20 (0.86; 1.67) |
|  |  |  |  |  |  |  |  |  |  | ̷⁄ | 1,365 (7.7) | 103 | 1.41 (1.02; 1.97) |
|  |  |  |  |  | ⁄ | 5,842 (32.8) | 293 | 0.99 (0.83; 1.17) |  | ─ | 1,691 (9.5) | 94 | 1.28 (0.92; 1.79) |
|  |  |  |  |  |  |  |  |  |  | ⁄ | 2,786 (15.6) | 96 | 1.06 (0.76; 1.49) |
|  |  |  |  |  |  |  |  |  |  | ̷⁄ | 278 (1.6) | 77 | 2.01 (1.41; 2.86) |
|  |  |  |  |  | ⁄ | 1,294 (7.3) | 250 | 1.36 (1.13; 1.64) |  | ─ | 272 (1.5) | 48 | 1.51 (1.02; 2.24) |
|  |  |  |  |  |  |  |  |  |  | ⁄ | 744 (4.2) | 125 | 1.70 (1.23; 2.34) |
|  |  |  |  |  |  |  |  |  |  | ̷⁄ | 323 (1.8) | 67 | 1.65 (1.15; 2.37) |
| 25.0 – 29.9 kg/m^2^ | 4,958 (27.8) | 684 | 1.18 (1.06; 1.31) |  | ─ | 1,307 (7.3) | 185 | 1.14 (0.94; 1.39) |  | ─ | 322 (1.8) | 44 | 1.27 (0.85; 1.89) |
|  |  |  |  |  |  |  |  |  |  | ⁄ | 662 (3.7) | 74 | 1.42 (1.001; 2.02) |
|  |  |  |  |  |  |  |  |  |  | ̷⁄ | 721 (4.0) | 108 | 1.82 (1.31; 2.52) |
|  |  |  |  |  | ⁄ | 2,357 (13.2) | 249 | 1.24 (1.03; 1.49) |  | ─ | 545 (3.1) | 51 | 1.27 (0.87; 1.88) |
|  |  |  |  |  |  |  |  |  |  | ⁄ | 1,091 (6.1) | 90 | 1.50 (1.07; 2.10) |
|  |  |  |  |  |  |  |  |  |  | ̷⁄ | 200 (1.1) | 66 | 3.08 (2.14; 4.44) |
|  |  |  |  |  | ⁄ | 657 (3.7) | 151 | 1.90 (1.54; 2.35) |  | ─ | 117 (0.7) | 29 | 2.43 (1.54; 3.84) |
|  |  |  |  |  |  |  |  |  |  | ⁄ | 340 (1.9) | 56 | 1.93 (1.33; 2.80) |
|  |  |  |  |  |  |  |  |  |  | ̷⁄ | 120 (0.7) | 27 | 2.20 (1.38; 3.50) |
| ≥30.0 kg/m^2^ | 1,812 (10.2) | 295 | 1.52 (1.32; 1.75) |  | ─ | 351 (2.0) | 57 | 1.36 (1.02; 1.83) |  | ─ | 71 (0.4) | 9 | 1.06 (0.52; 2.15) |
|  |  |  |  |  |  |  |  |  |  | ⁄ | 160 (0.9) | 21 | 1.73 (1.05; 2.87) |
|  |  |  |  |  |  |  |  |  |  | ̷⁄ | 294 (1.7) | 39 | 1.87 (1.24; 2.83) |
|  |  |  |  |  | ⁄ | 804 (4.5) | 87 | 1.40 (1.09; 1.79) |  | ─ | 160 (0.9) | 16 | 1.61 (0.92; 2.83) |
|  |  |  |  |  |  |  |  |  |  | ⁄ | 350 (2.0) | 32 | 1.72 (1.11; 2.66) |

* change of BMI (<-0.10 kg/m²/year, -0.10 – 0.09 kg/m²/year, ≥0.10 kg/m²/year) between baseline and year 5 (t_0-5_) and between year 5 and 10 (t_5-10_)

^§^ Hazard rate ratio (HRR) and 95% confidence interval (95% CI) adjusted for smoking status and stratified for age

^#^ reference category
